# Supplementary material for: Assessing the effectiveness of COVID-19 vaccine lotteries: A cross-state synthetic control methods approach
Source: PLoS One. 2022 Sep 28;17(9):e0274374. doi: 10.1371/journal.pone.0274374 (PMC9518920; doi:10.1371/journal.pone.0274374)
Supplement: S1 Appendix — (PDF) [file pone.0274374.s001.pdf]

Appendix S1

Optimization Functions

Optimization was performed using all available methods in the R package optimx with the best-performing method's results being used. For more details on these specific methods, see pg.47–48 of optimx's description file. These methods include:

- Nelder-Mead
- BFGS
- CG
- L-BFGS-B
- nlm
- nlminb
- spg
- ucminf
- newuoa
- bobyqa
- nmkb
- hjkb
- Rcgmin
- Rvmmin

We also set our ipop optimization options to:

```
margin_ipop = .02, (how close we get to the constraints)
sigf_ipop = 7, (the precision required, 7 significant figures)
bound_ipop = 6 (clipping bound for the variables)
```
