# Supplementary material for: Assessing the effectiveness of COVID-19 vaccine lotteries: A cross-state synthetic control methods approach
Source: PLoS One. 2022 Sep 28;17(9):e0274374. doi: 10.1371/journal.pone.0274374 (PMC9518920; doi:10.1371/journal.pone.0274374)
Supplement: S2 Appendix — (PDF) [file pone.0274374.s002.pdf]

Appendix S2

Observed and Synthetic Vaccination Trend Lines by States with Lotteries (First-Dose)

AR

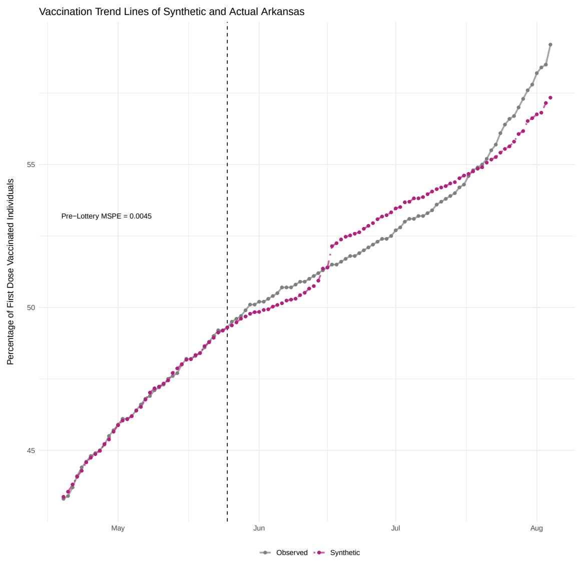

CA

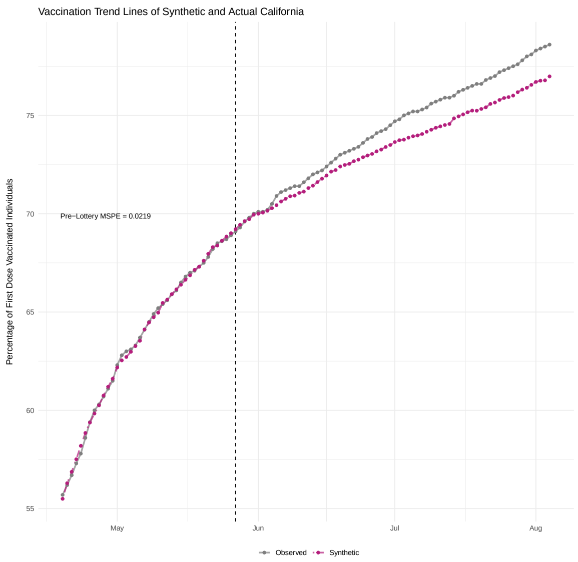

CO

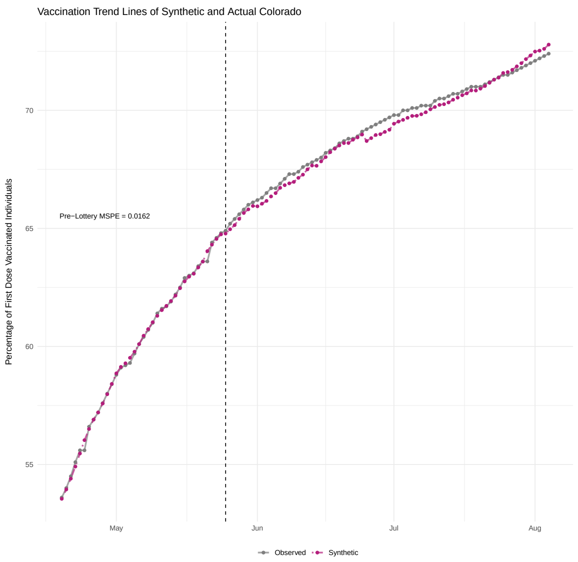

DE

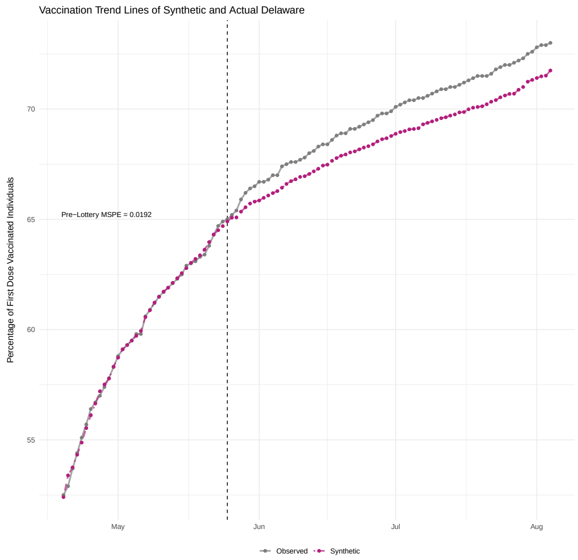

IL

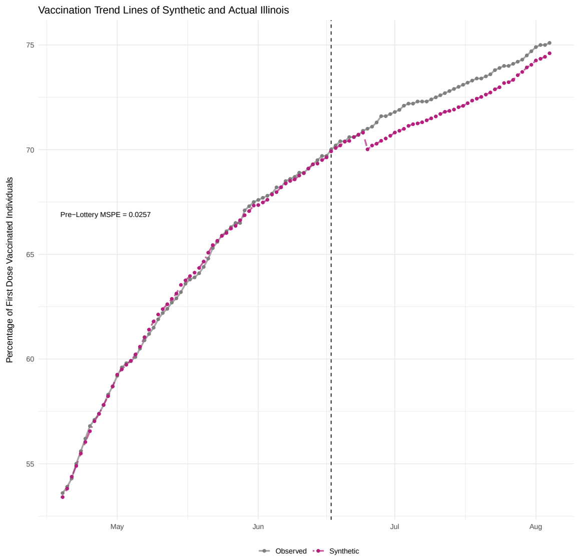

KY

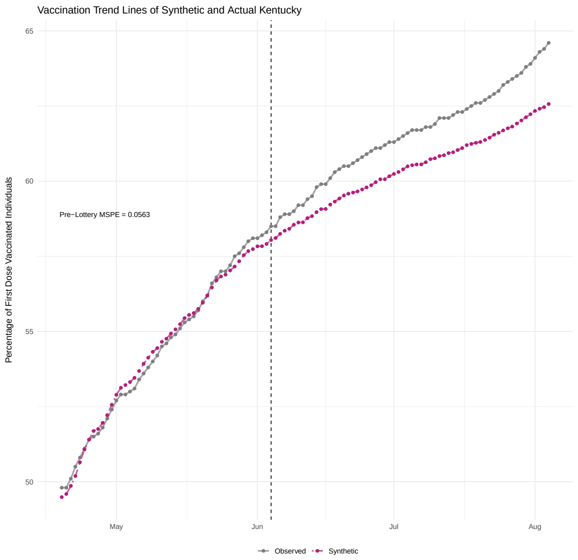

LA

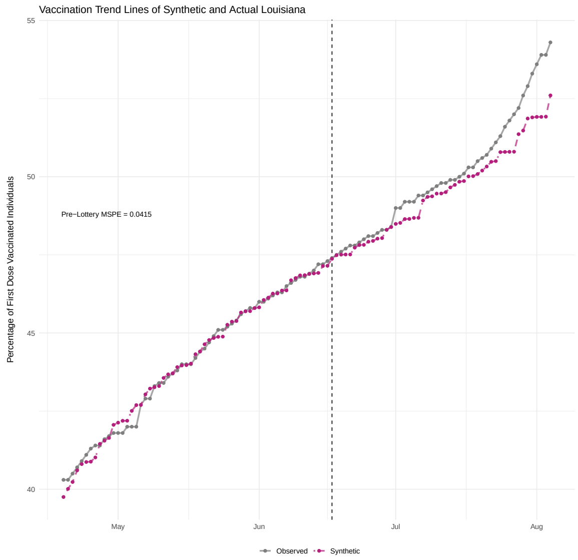

MA

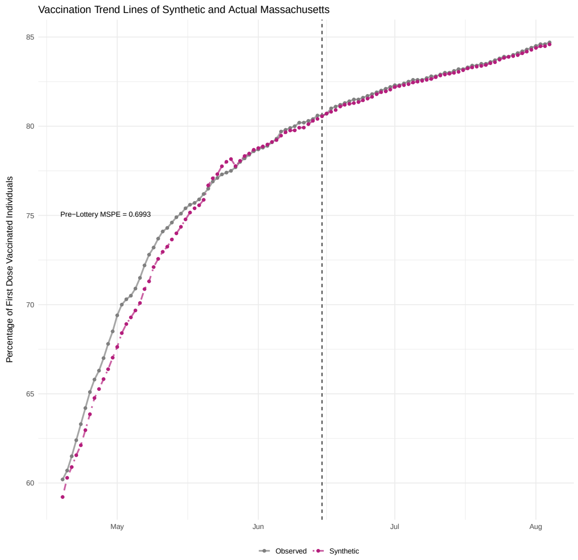

MD

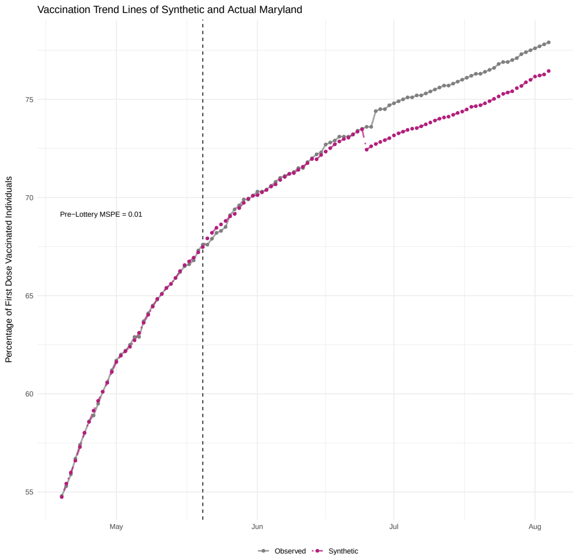

ME

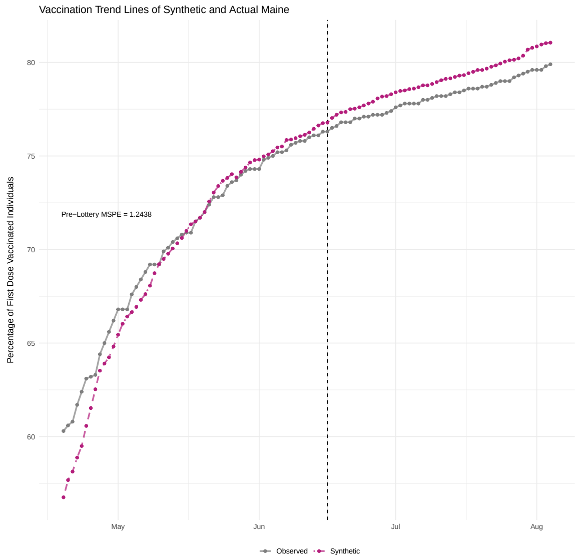

MI

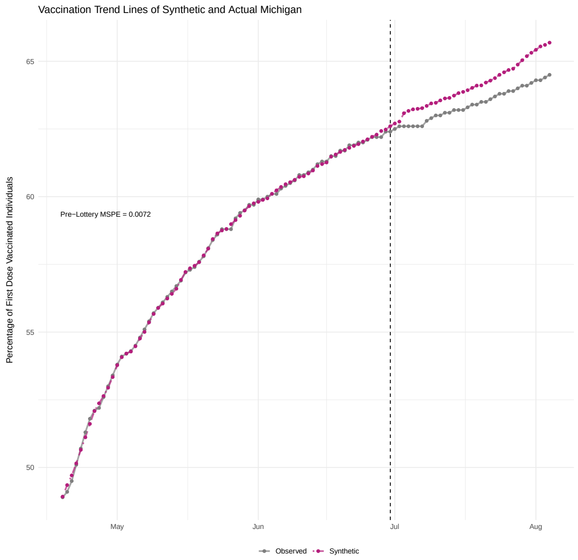

MO

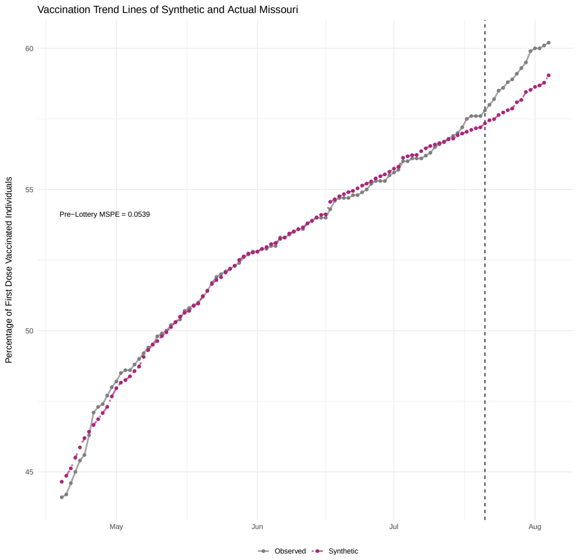

NC

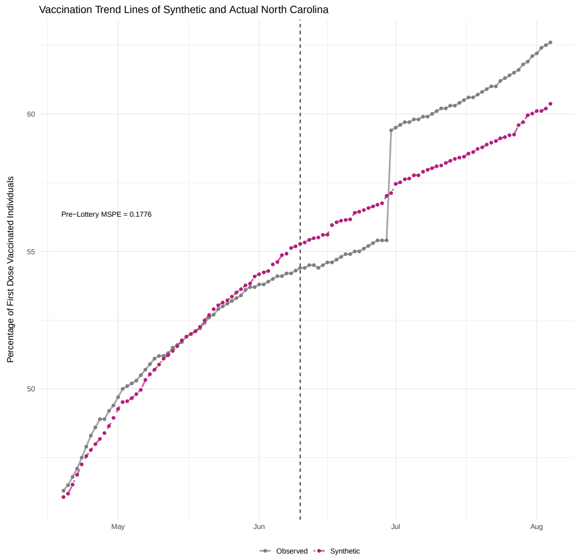

NM

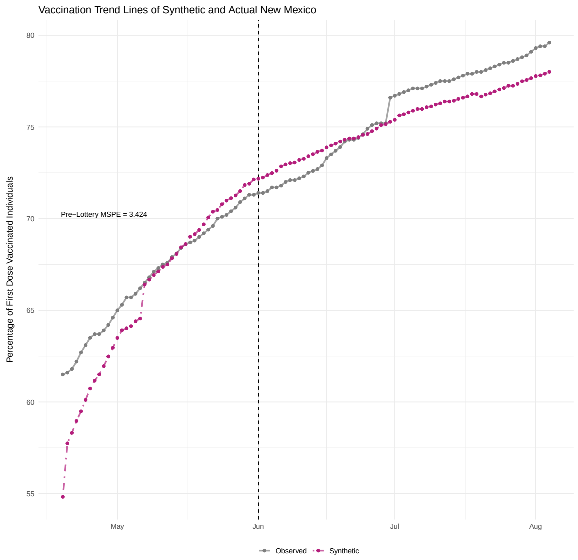

NV

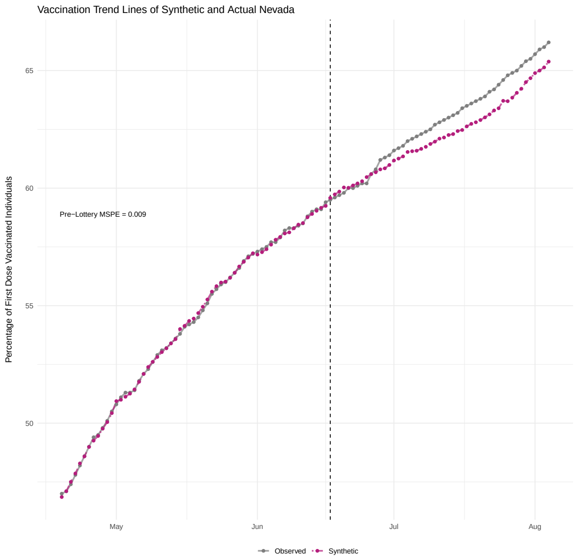

NY

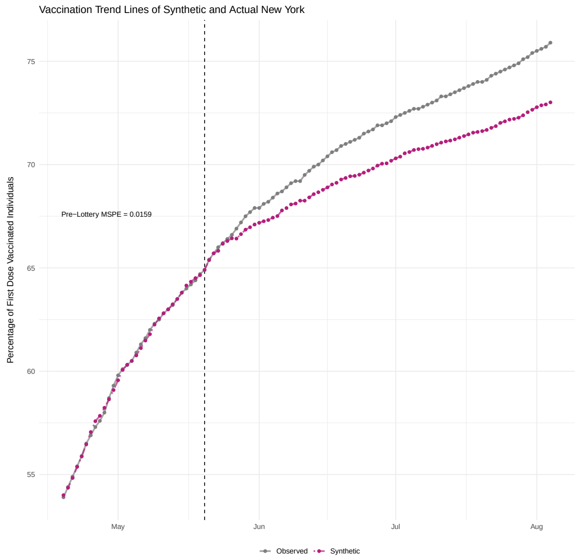

OH

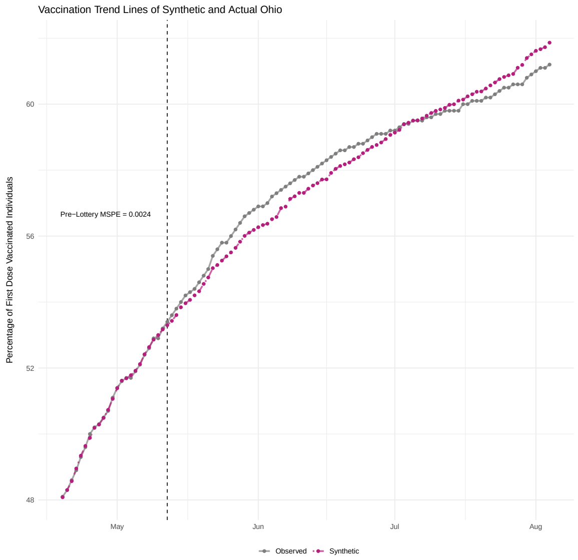

OR

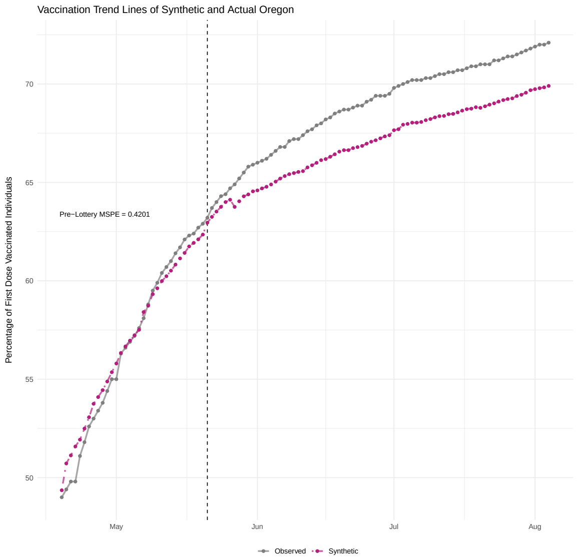

WA

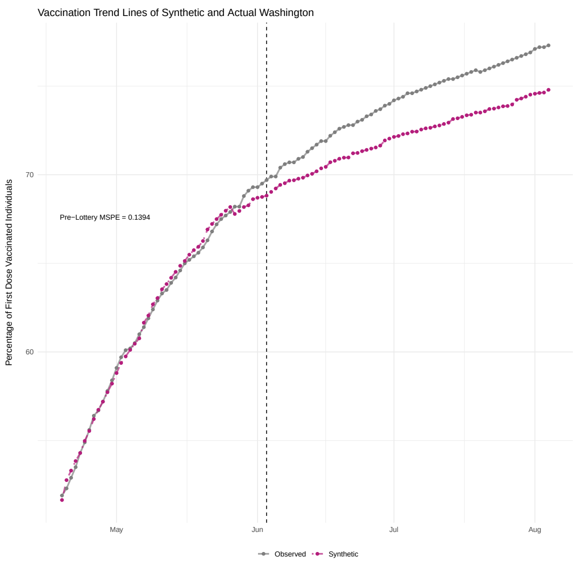

Vaccination Trend Lines of Synthetic and Actual Washington
Percentage of First Dose Vaccinated Individuals
70
60
Pre−Lottery MSPE = 0.1394
May
Jun
Jul
Aug
Observed
Synthetic

WV

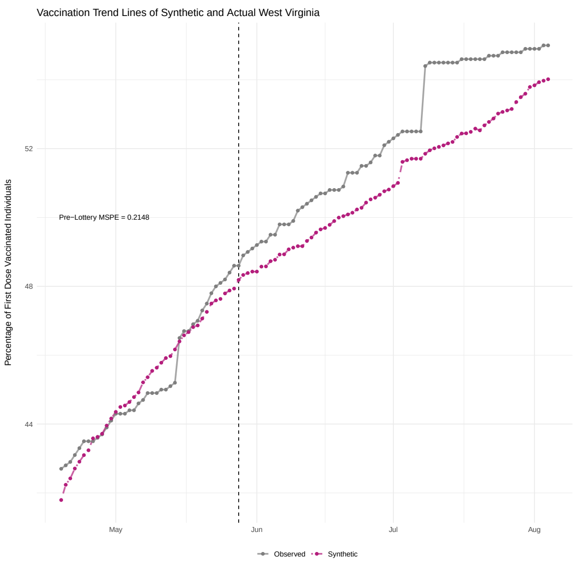

Vaccination Trend Lines of Synthetic and Actual West Virginia
Percentage of First Dose Vaccinated Individuals
52
48
44
Pre−Lottery MSPE = 0.2148
May
Jun
Jul
Aug
Observed
Synthetic

Observed and Synthetic Vaccination Trend Lines by States with Lotteries (Complete)

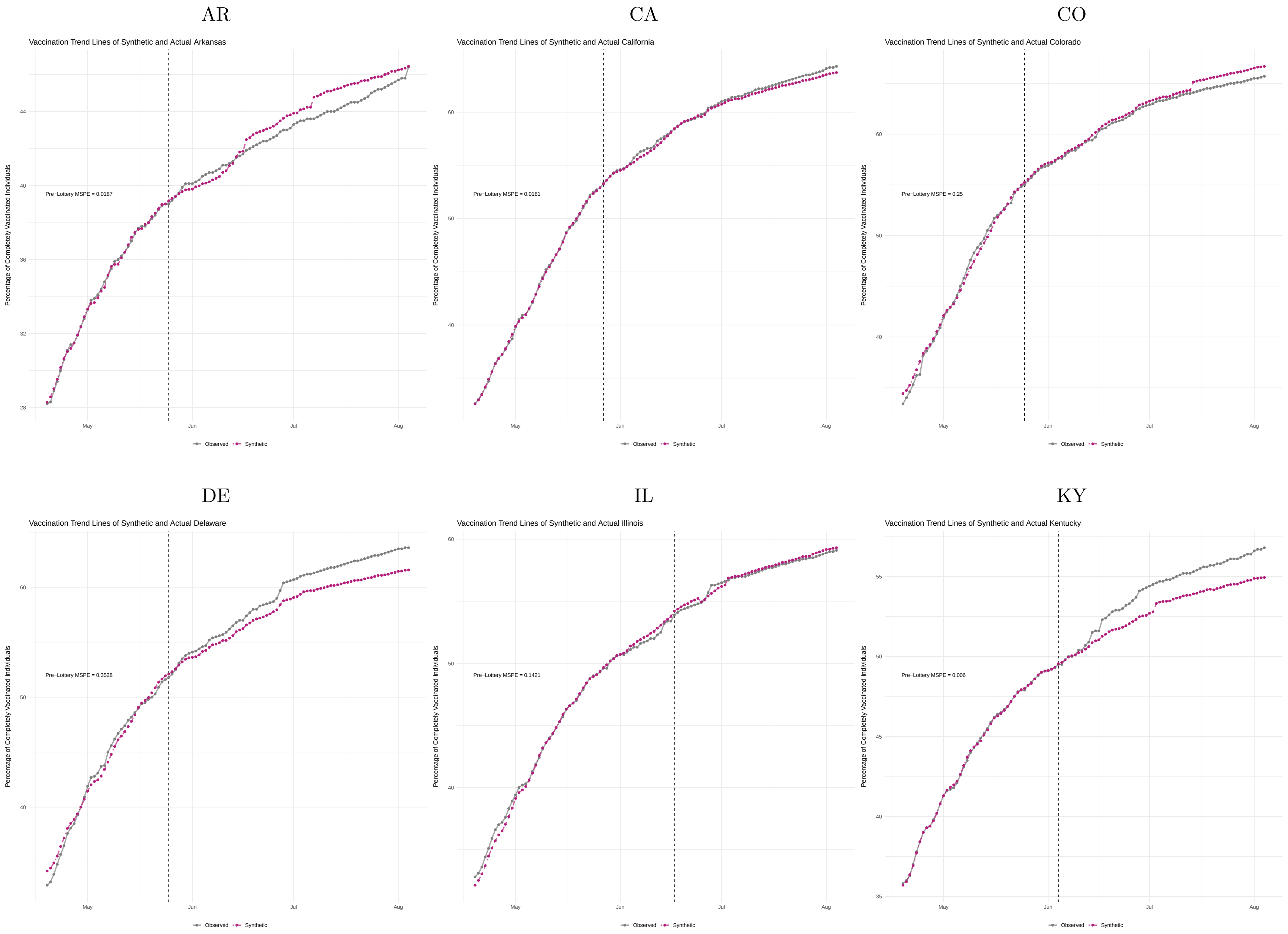

LA

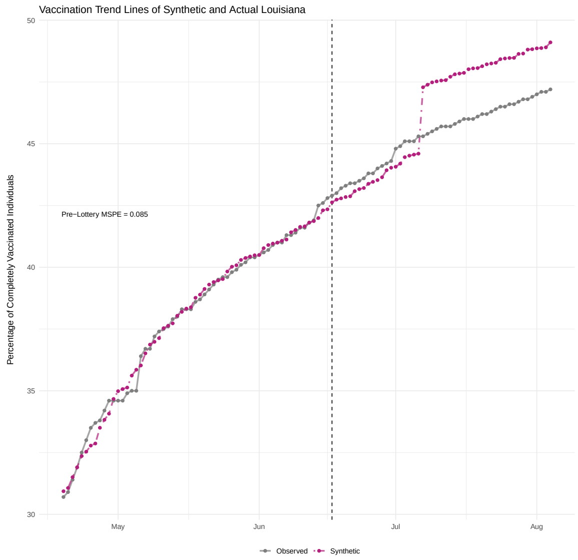

MA

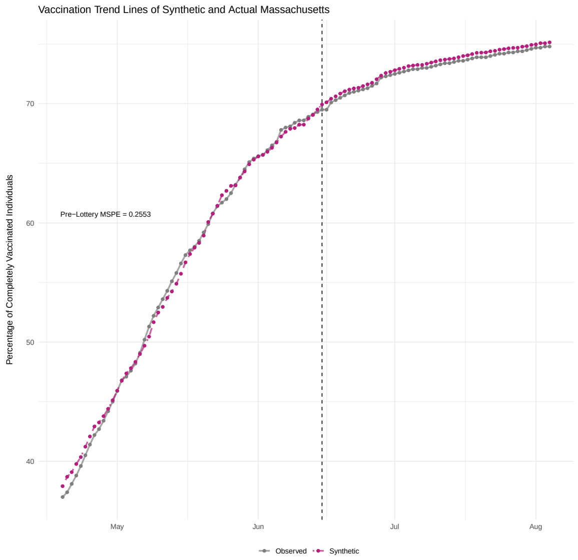

MD

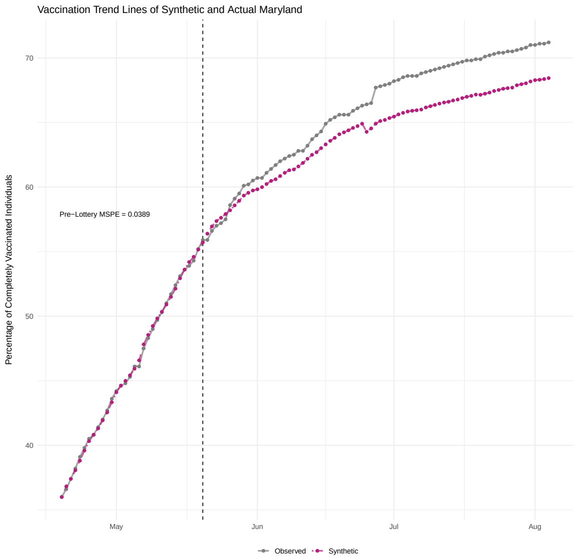

ME

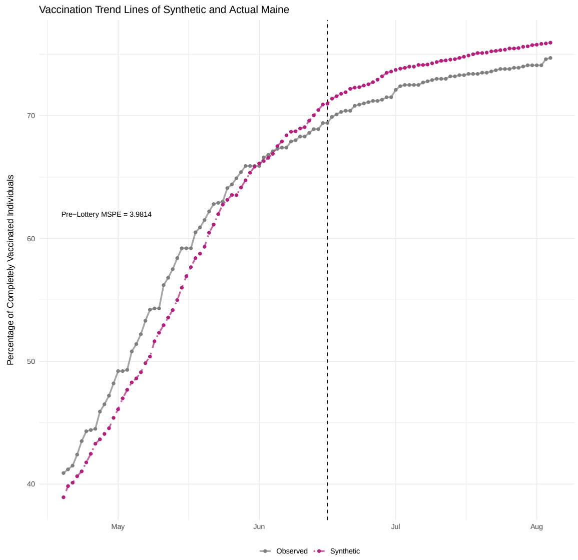

MI

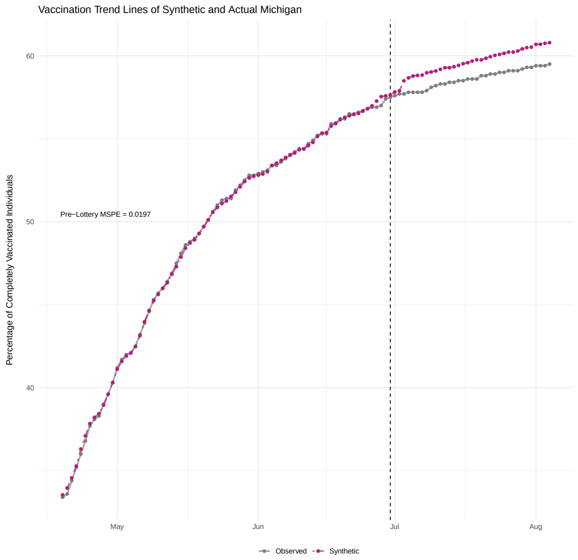

MO

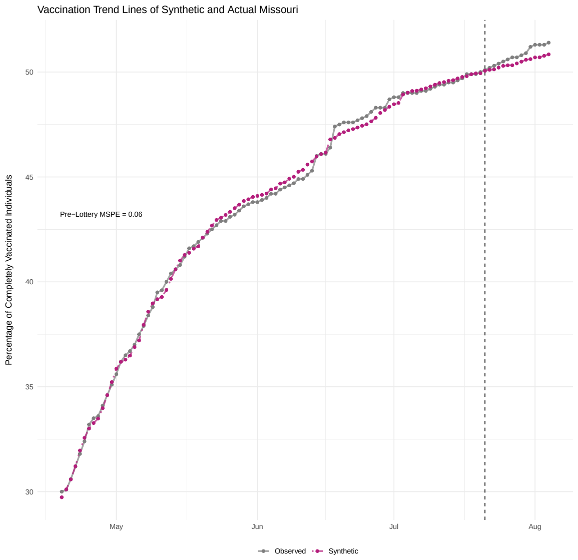

NC

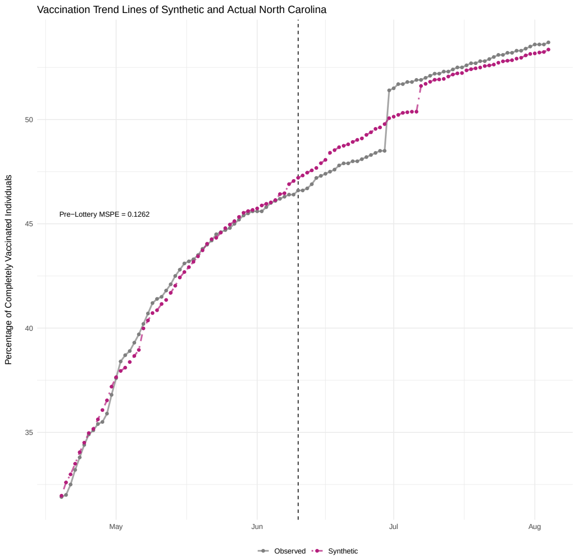

NM

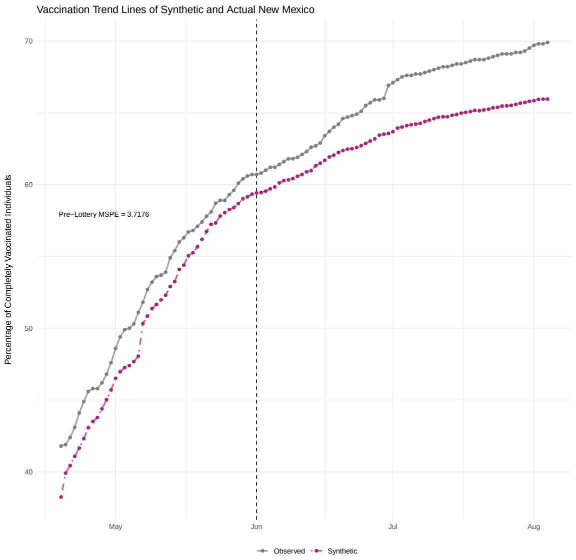

NV

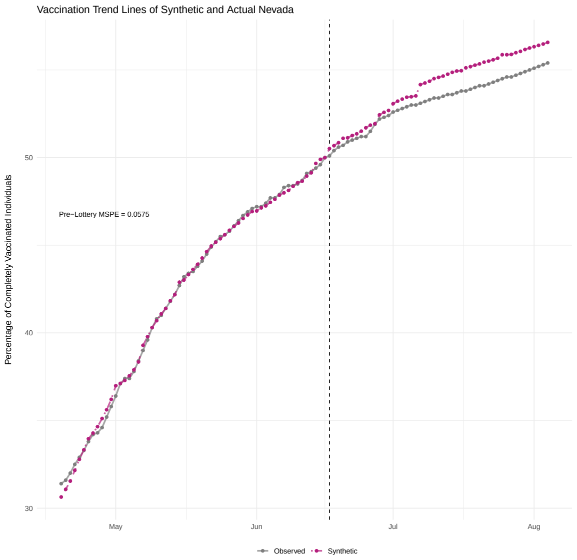

NY

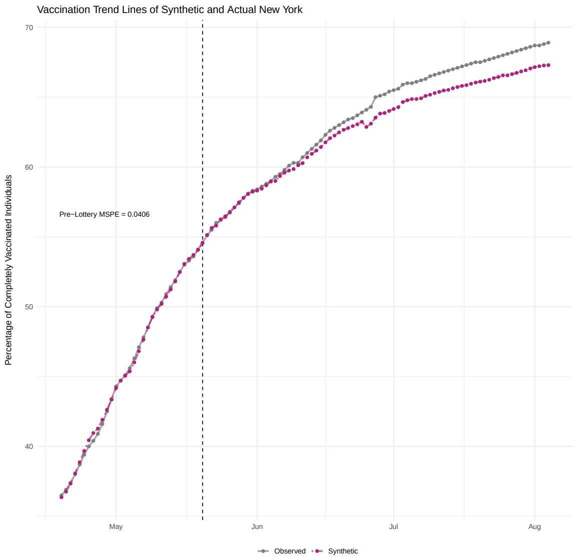

OH

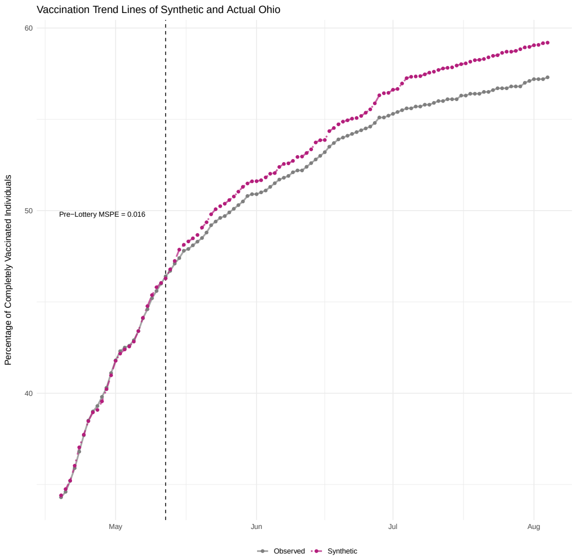

OR

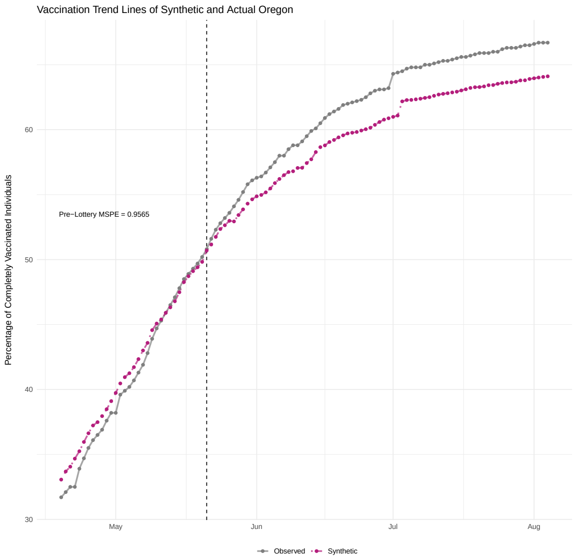

WA

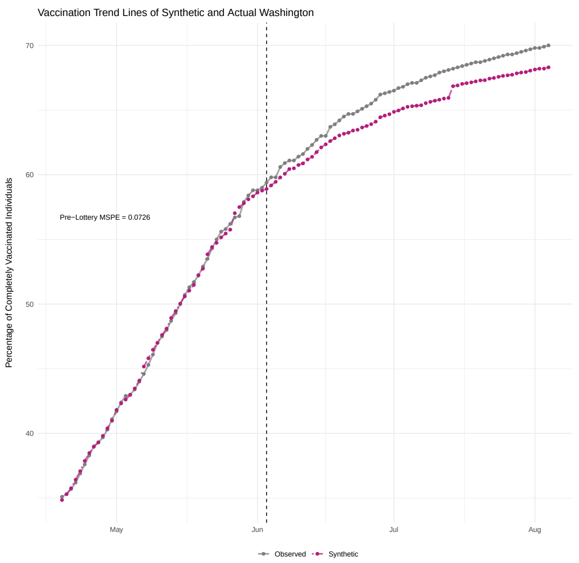
Vaccination Trend Lines of Synthetic and Actual Washington
Percentage of Completely Vaccinated Individuals
70
60
50
40
Pre−Lottery MSPE = 0.0726
May
Jun
Jul
Aug
Observed
Synthetic

WV

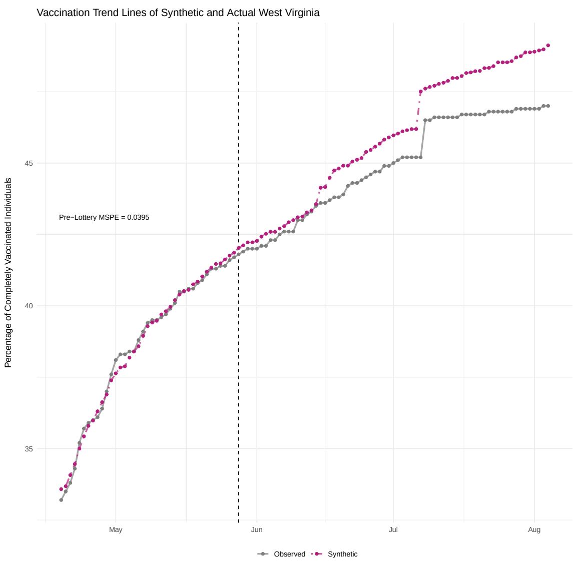
Vaccination Trend Lines of Synthetic and Actual West Virginia
Percentage of Completely Vaccinated Individuals
45
40
35
Pre−Lottery MSPE = 0.0395
May
Jun
Jul
Aug
Observed
Synthetic

Synthetic Control Weights (First-Dose)

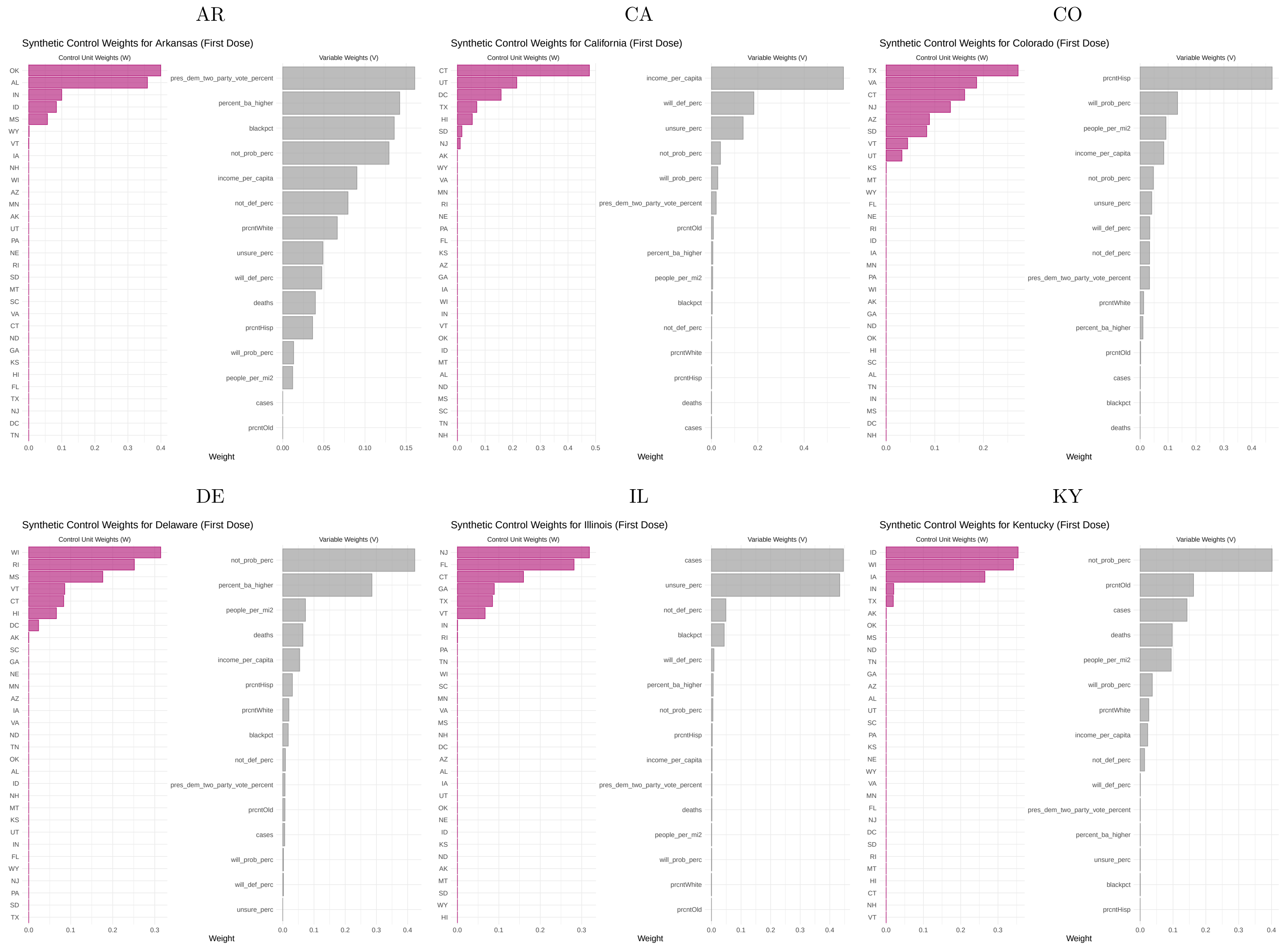

LA

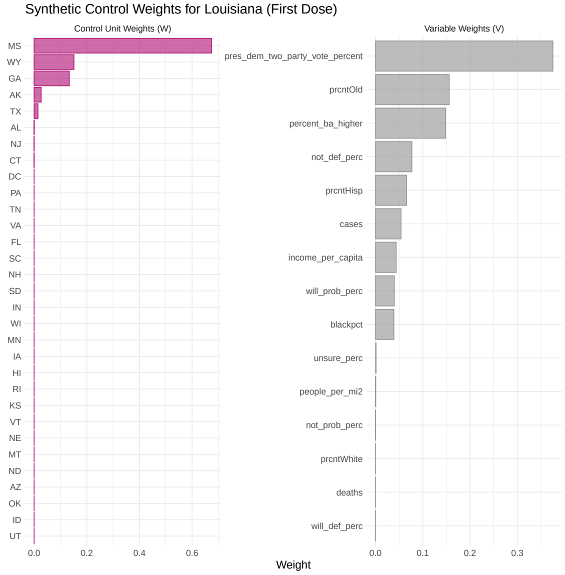

MA

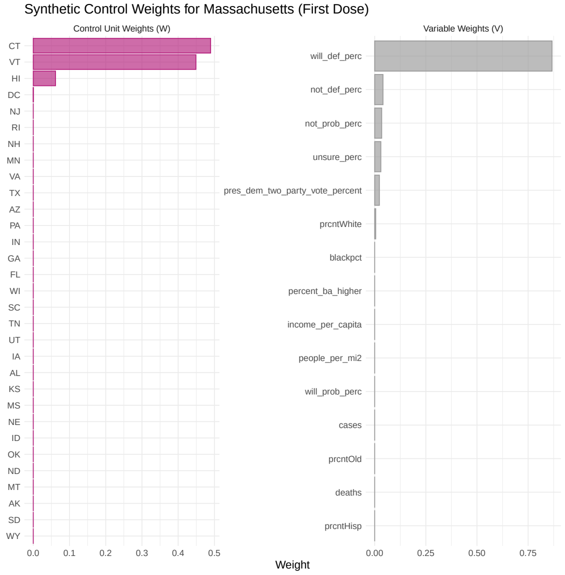

MD

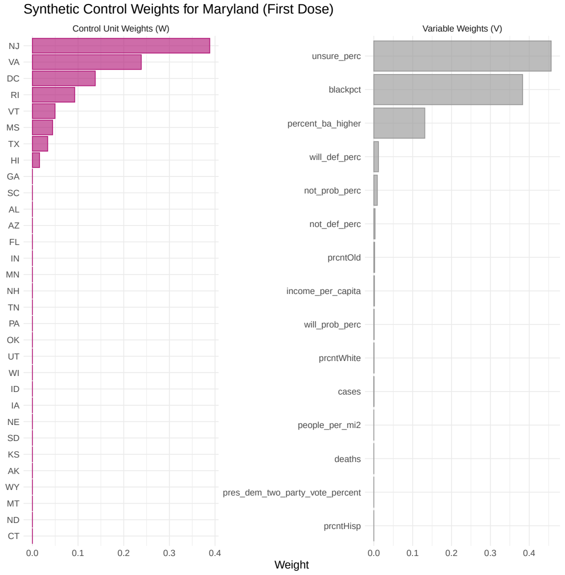

ME

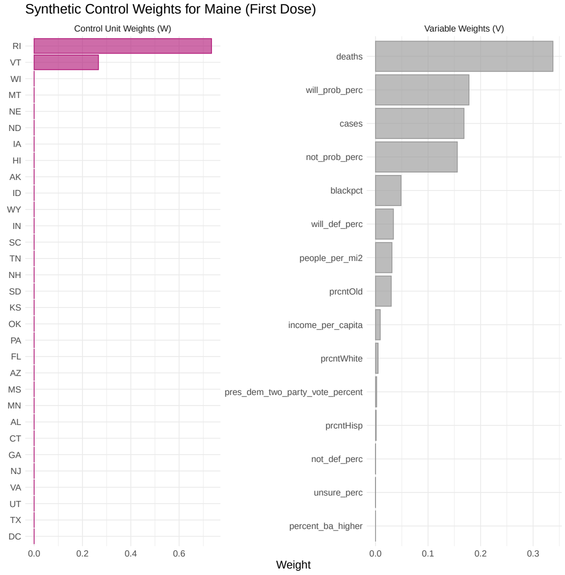

MI

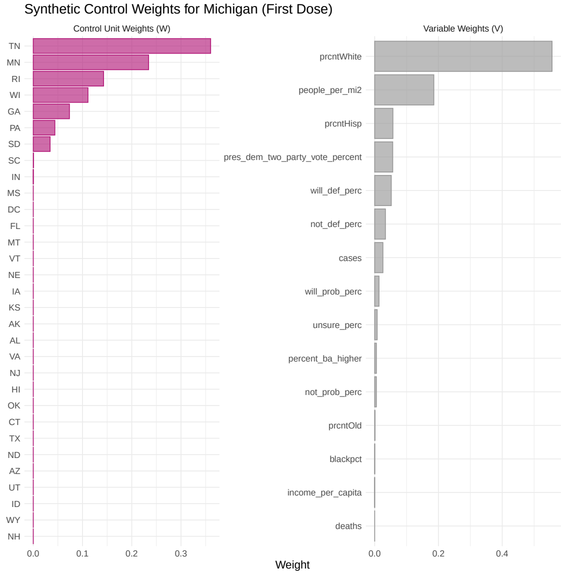

MO

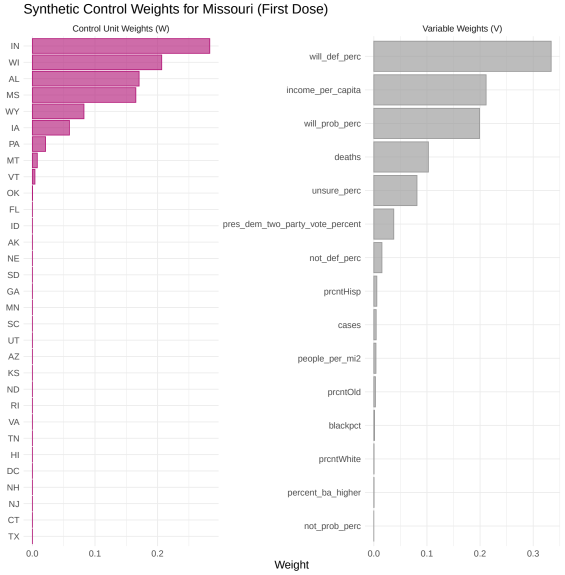

NC

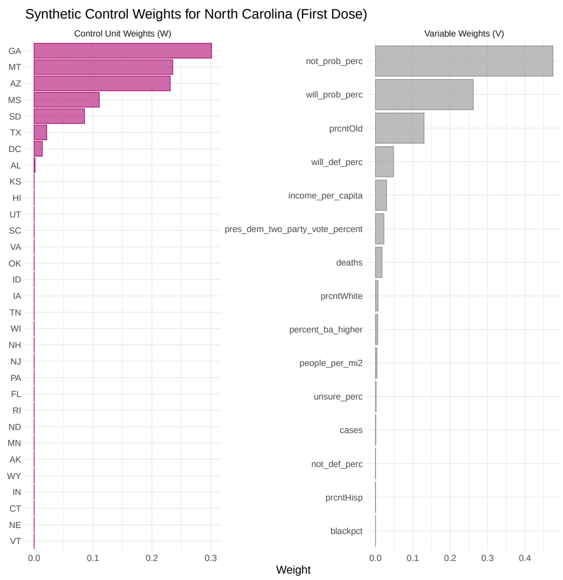

NM

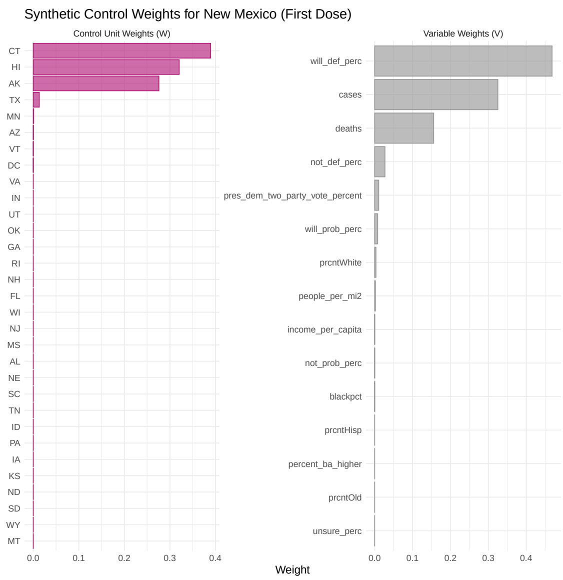

NV

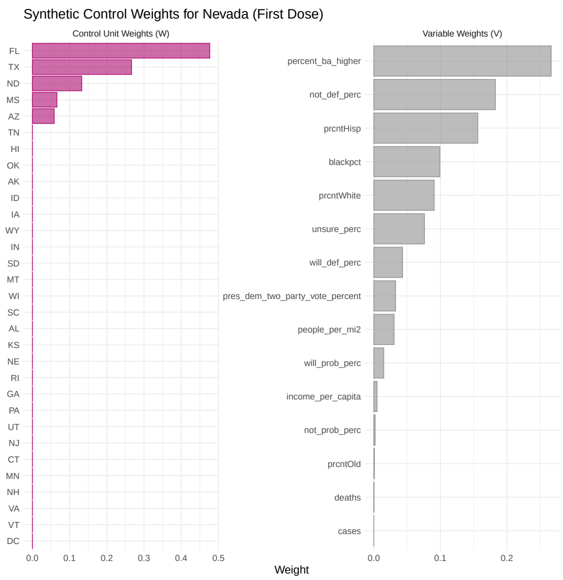

NY

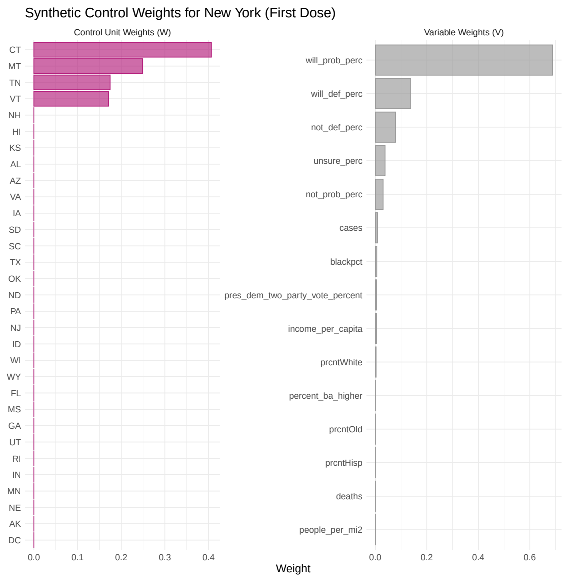

OH

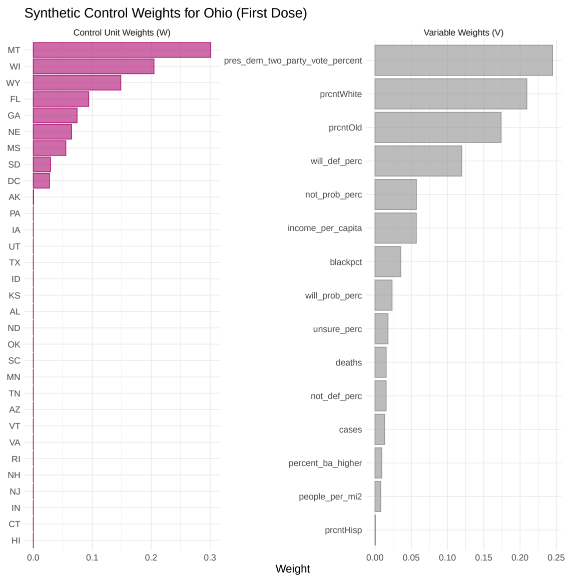

OR

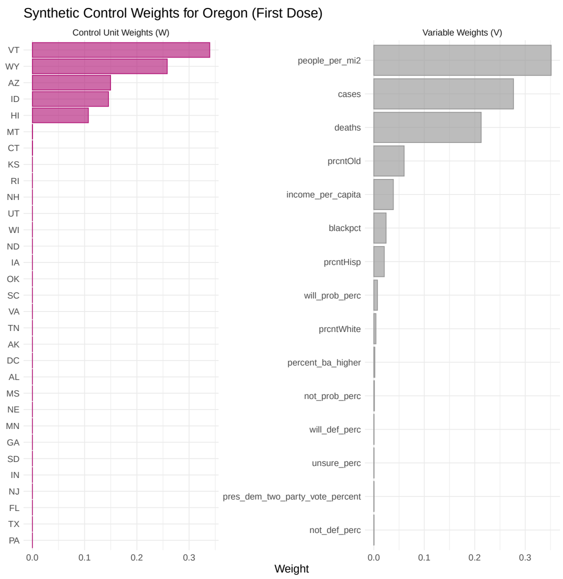

WA

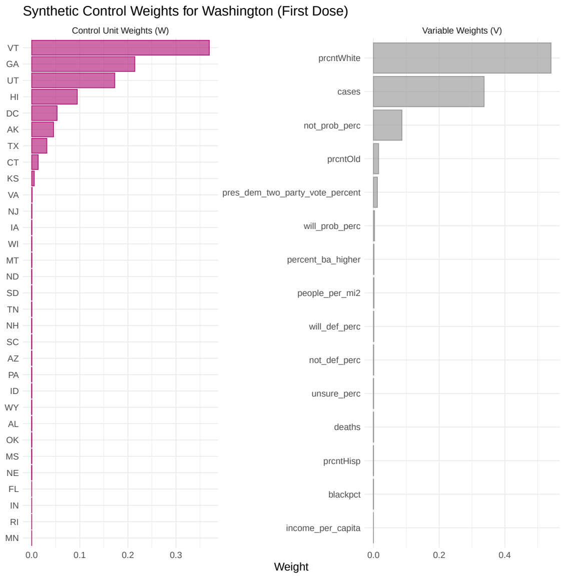

Synthetic Control Weights for Washington (First Dose)
Control Unit Weights (W)
VT
GA
UT
HI
DC
AK
TX
CT
KS
VA
NJ
IA
WI
MT
ND
SD
TN
NH
SC
AZ
PA
ID
WY
AL
OK
MS
NE
FL
IN
RI
MN
0.0
0.1
0.2
0.3
Variable Weights (V)
prcntWhite
cases
not_prob_perc
prcntOld
pres_dem_two_party_vote_percent
will_prob_perc
percent_ba_higher
people_per_mi2
will_def_perc
not_def_perc
unsure_perc
deaths
prcntHisp
blackpct
income_per_capita
0.0
0.2
0.4
Weight

WV

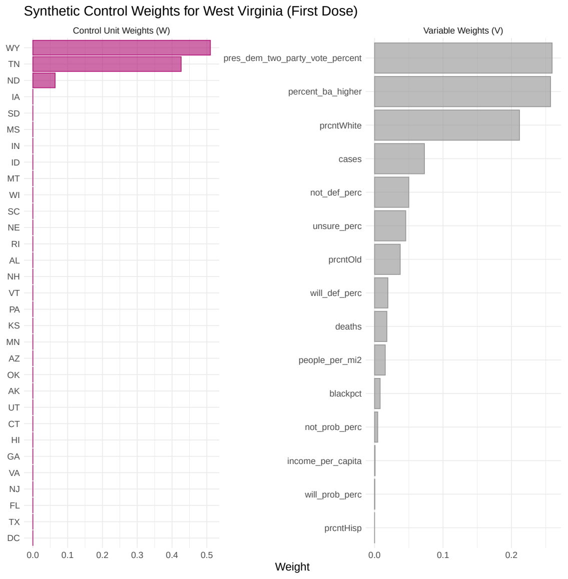

Synthetic Control Weights for West Virginia (First Dose)
Control Unit Weights (W)
WY
TN
ND
IA
SD
MS
IN
ID
MT
WI
SC
NE
RI
AL
NH
VT
PA
KS
MN
AZ
OK
AK
UT
CT
HI
GA
VA
NJ
FL
TX
DC
0.0
0.1
0.2
0.3
0.4
0.5
Variable Weights (V)
pres_dem_two_party_vote_percent
percent_ba_higher
prcntWhite
cases
not_def_perc
unsure_perc
prcntOld
will_def_perc
deaths
people_per_mi2
blackpct
not_prob_perc
income_per_capita
will_prob_perc
prcntHisp
0.0
0.1
0.2
Weight

Synthetic Control Weights (Complete)

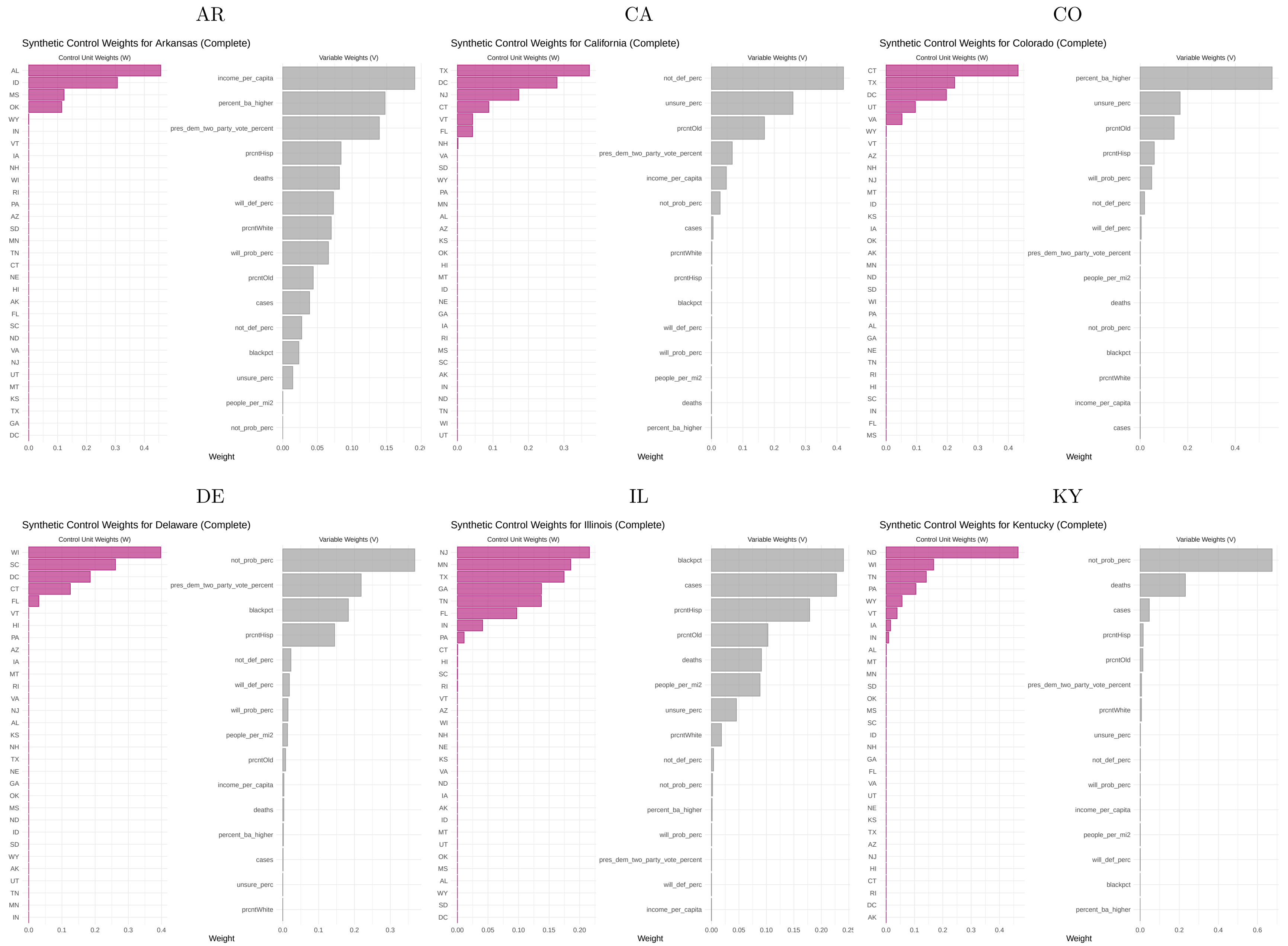

LA

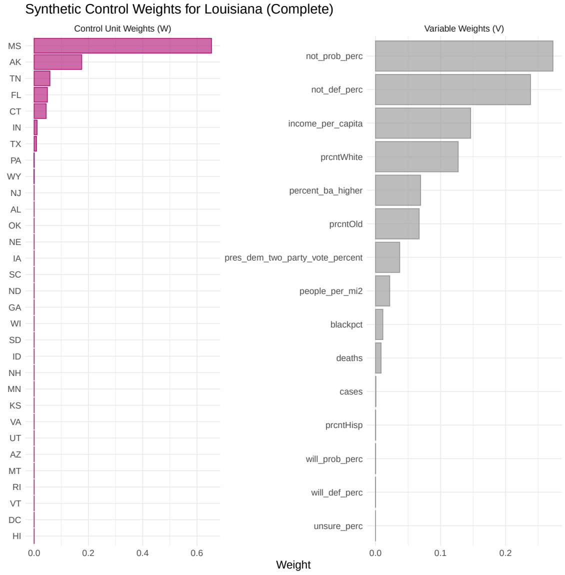

MA

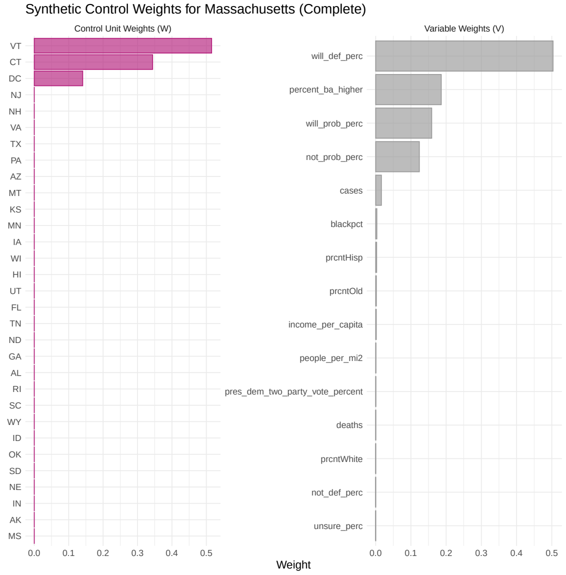

MD

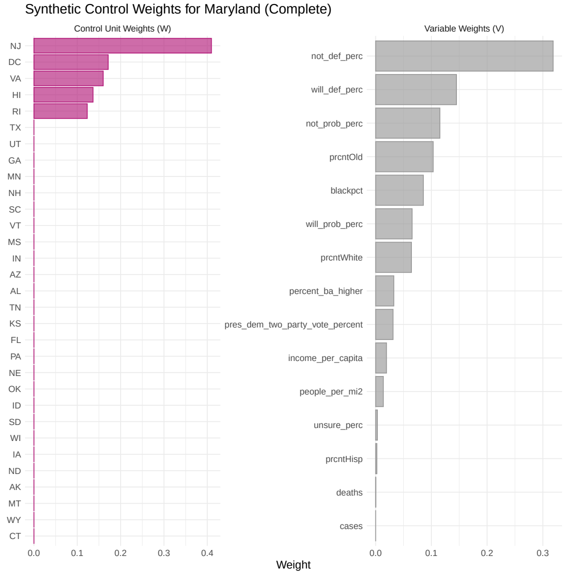

ME

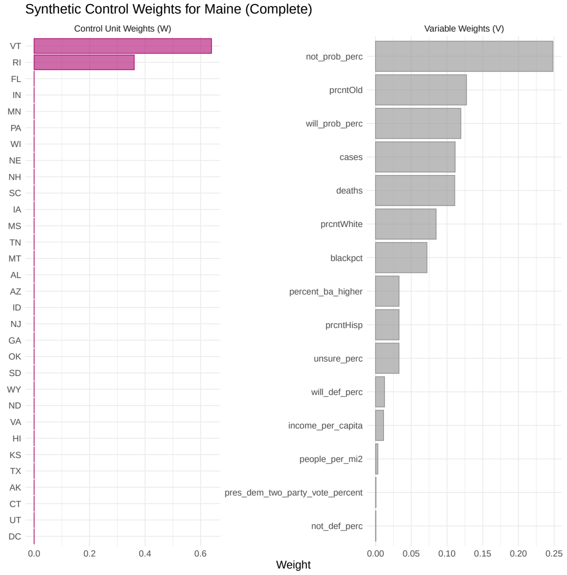

MI

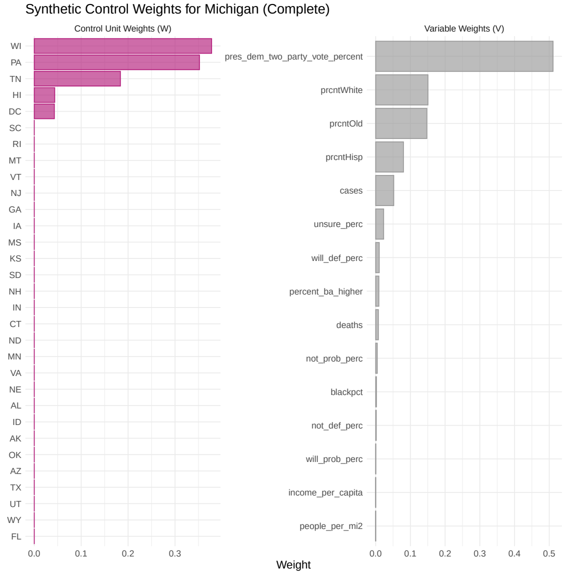

MO

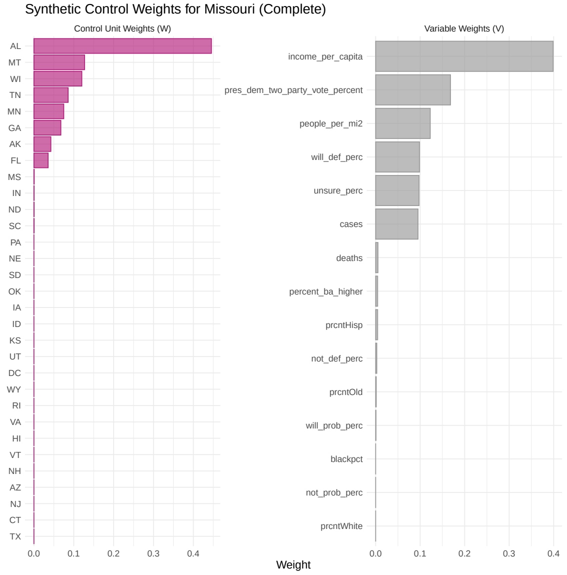

NC

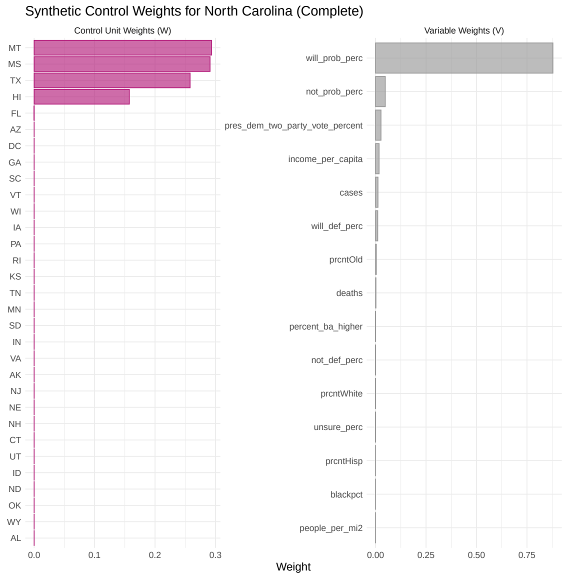

NM

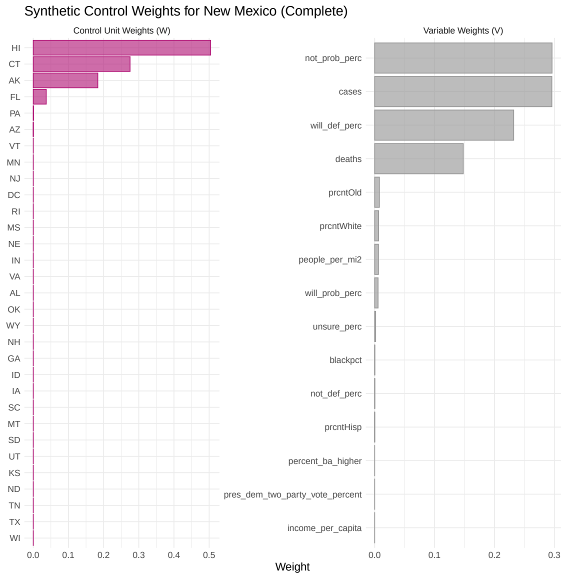

NV

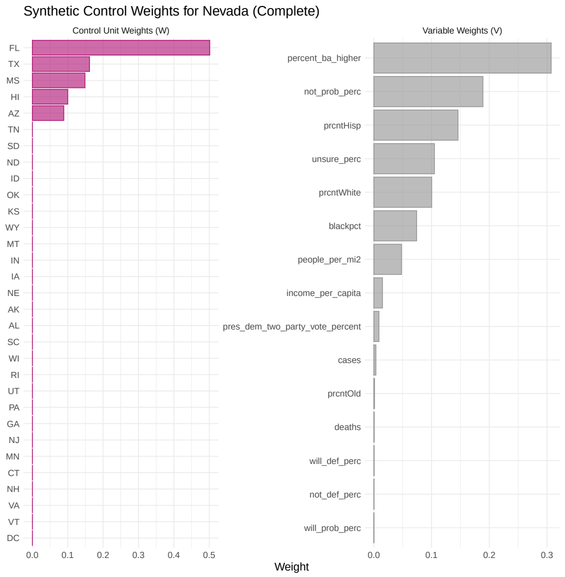

NY

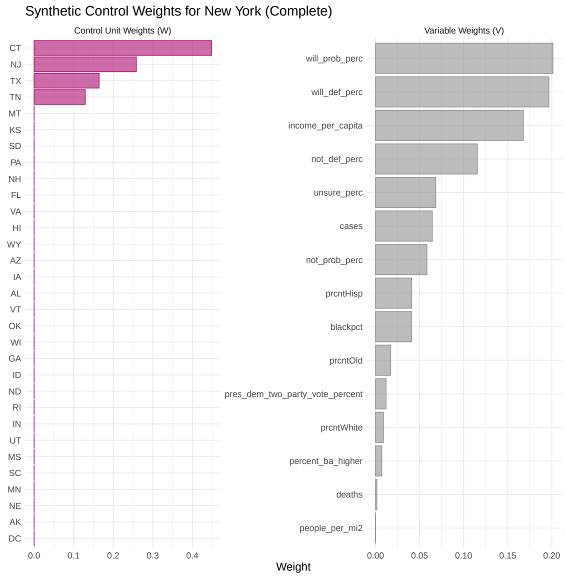

OH

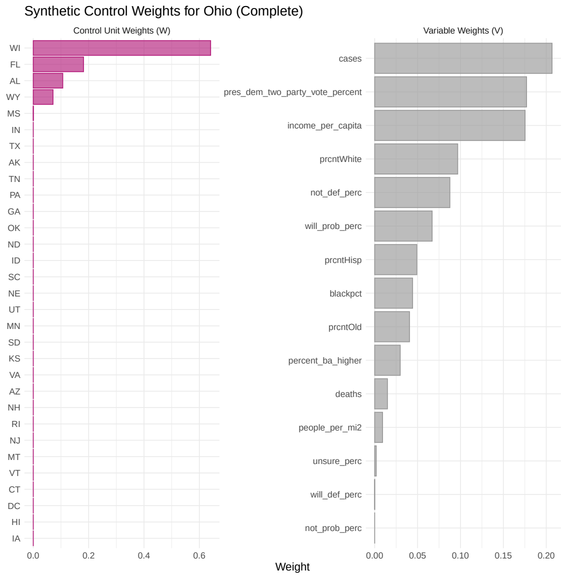

OR

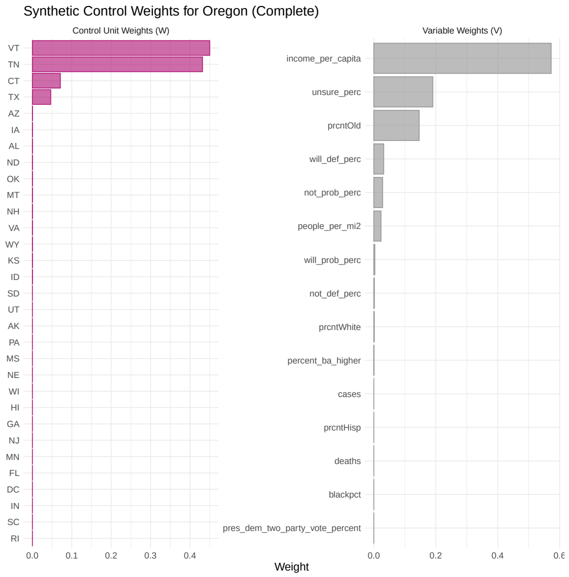

WA

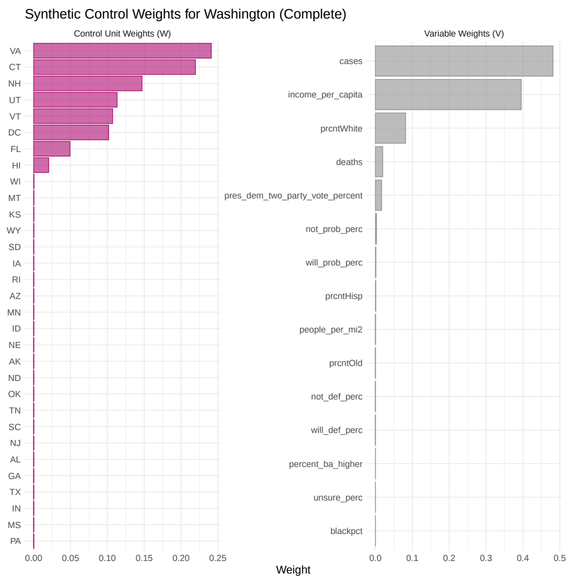
Synthetic Control Weights for Washington (Complete)
Control Unit Weights (W)
Variable Weights (V)
VA
CT
NH
UT
VT
DC
FL
HI
WI
MT
KS
WY
SD
IA
RI
AZ
MN
ID
NE
AK
ND
OK
TN
SC
NJ
AL
GA
TX
IN
MS
PA
cases
income_per_capita
prcntWhite
deaths
pres_dem_two_party_vote_percent
not_prob_perc
will_prob_perc
prcntHisp
people_per_mi2
prcntOld
not_def_perc
will_def_perc
percent_ba_higher
unsure_perc
blackpct
0.00 0.05 0.10 0.15 0.20 0.25
0.0 0.1 0.2 0.3 0.4 0.5
Weight

WV

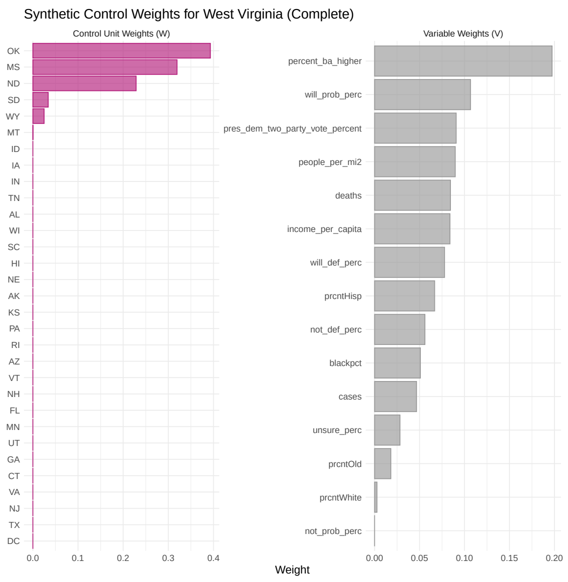
Synthetic Control Weights for West Virginia (Complete)
Control Unit Weights (W)
Variable Weights (V)
OK
MS
ND
SD
WY
MT
ID
IA
IN
TN
AL
WI
SC
HI
NE
AK
KS
PA
RI
AZ
VT
NH
FL
MN
UT
GA
CT
VA
NJ
TX
DC
percent_ba_higher
will_prob_perc
pres_dem_two_party_vote_percent
people_per_mi2
deaths
income_per_capita
will_def_perc
prcntHisp
not_def_perc
blackpct
cases
unsure_perc
prcntOld
prcntWhite
not_prob_perc
0.0 0.1 0.2 0.3 0.4
0.00 0.05 0.10 0.15 0.20
Weight
