# Supplementary figures and images for: Assessing the effectiveness of COVID-19 vaccine lotteries: A cross-state synthetic control methods approach
Source: PLoS One. 2022 Sep 28;17(9):e0274374. doi: 10.1371/journal.pone.0274374 (PMC9518920; doi:10.1371/journal.pone.0274374)

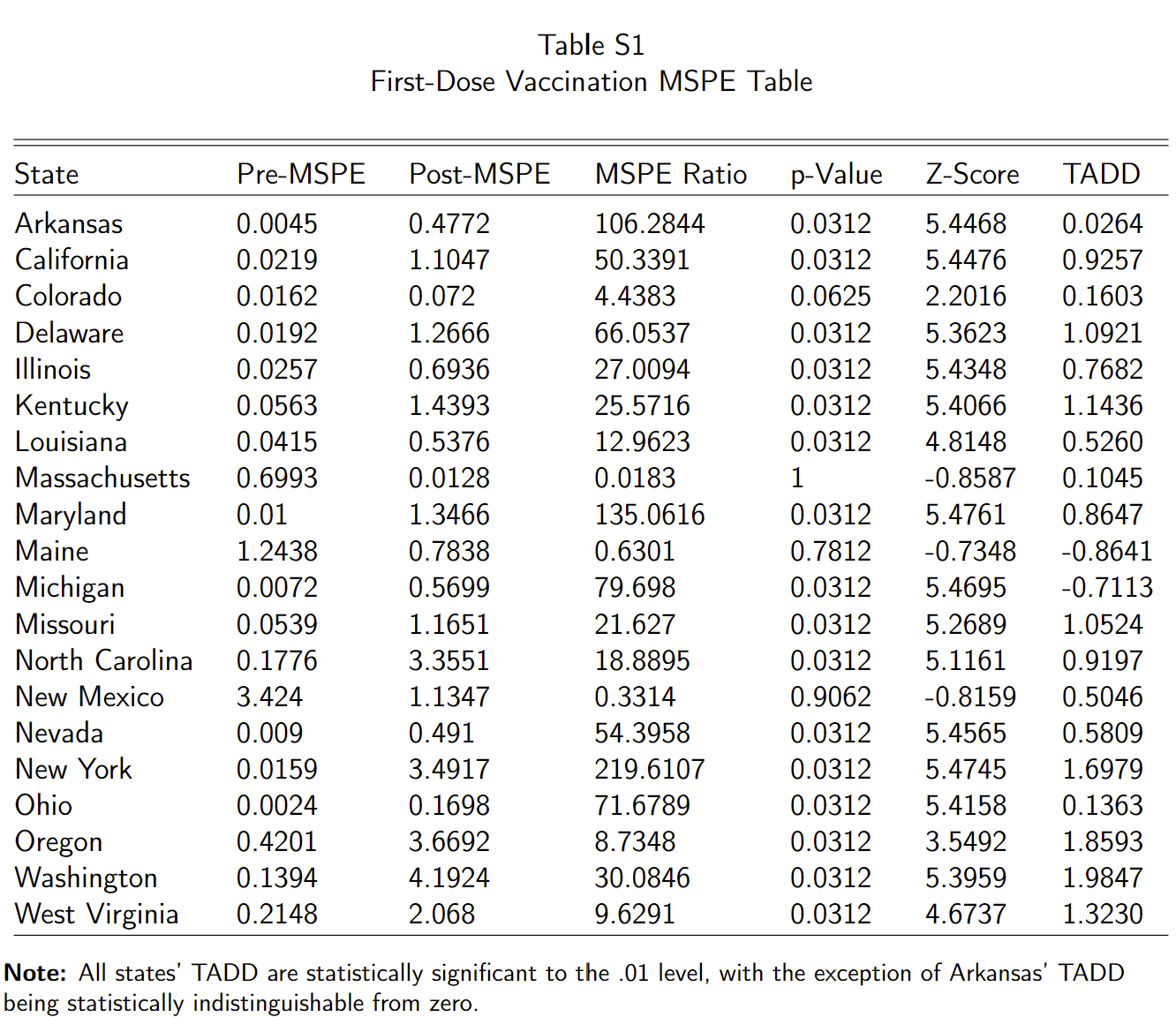

Supplement: S1 Table — (PNG) [file pone.0274374.s003.png]

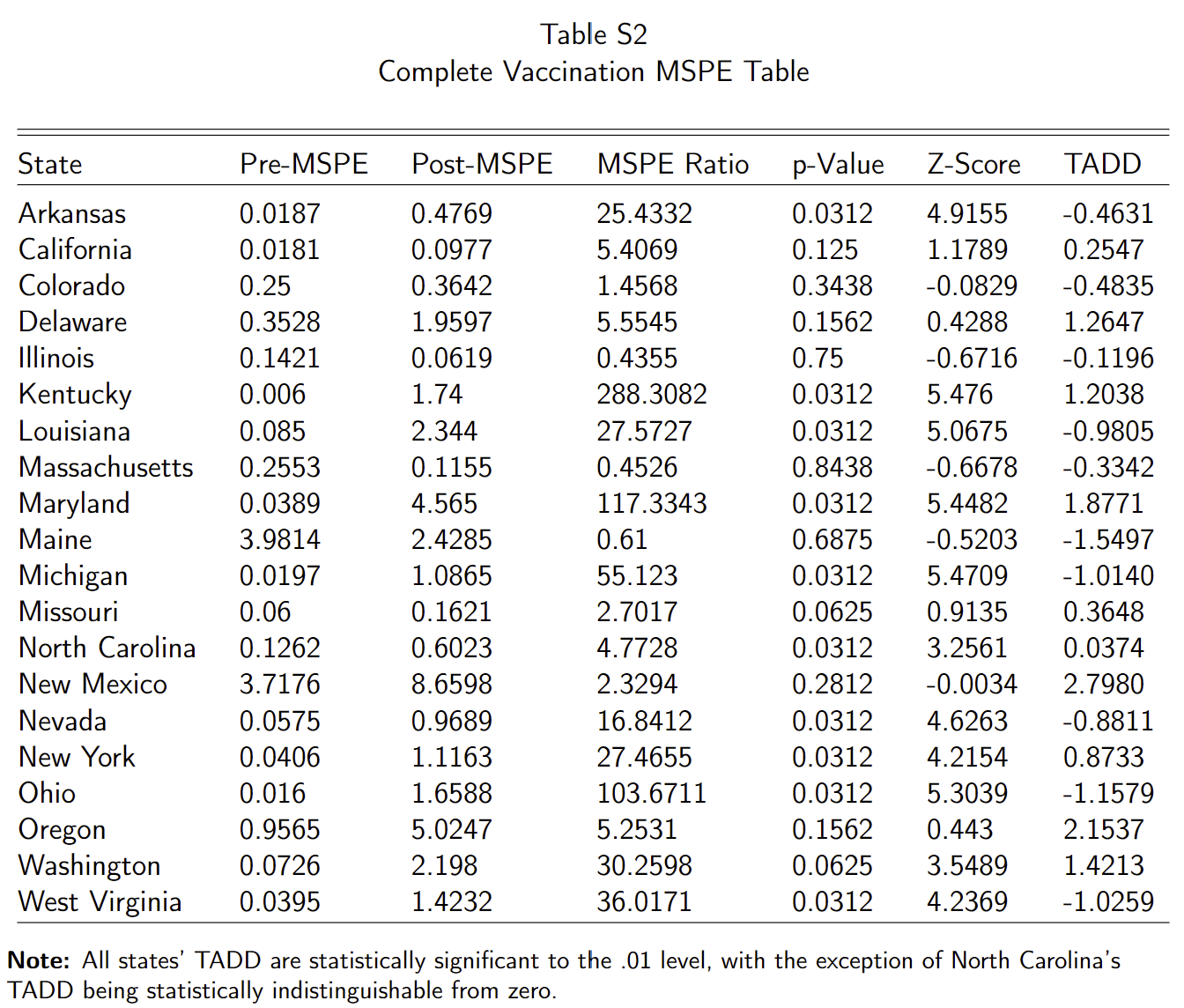

Supplement: S2 Table — (PNG) [file pone.0274374.s004.png]

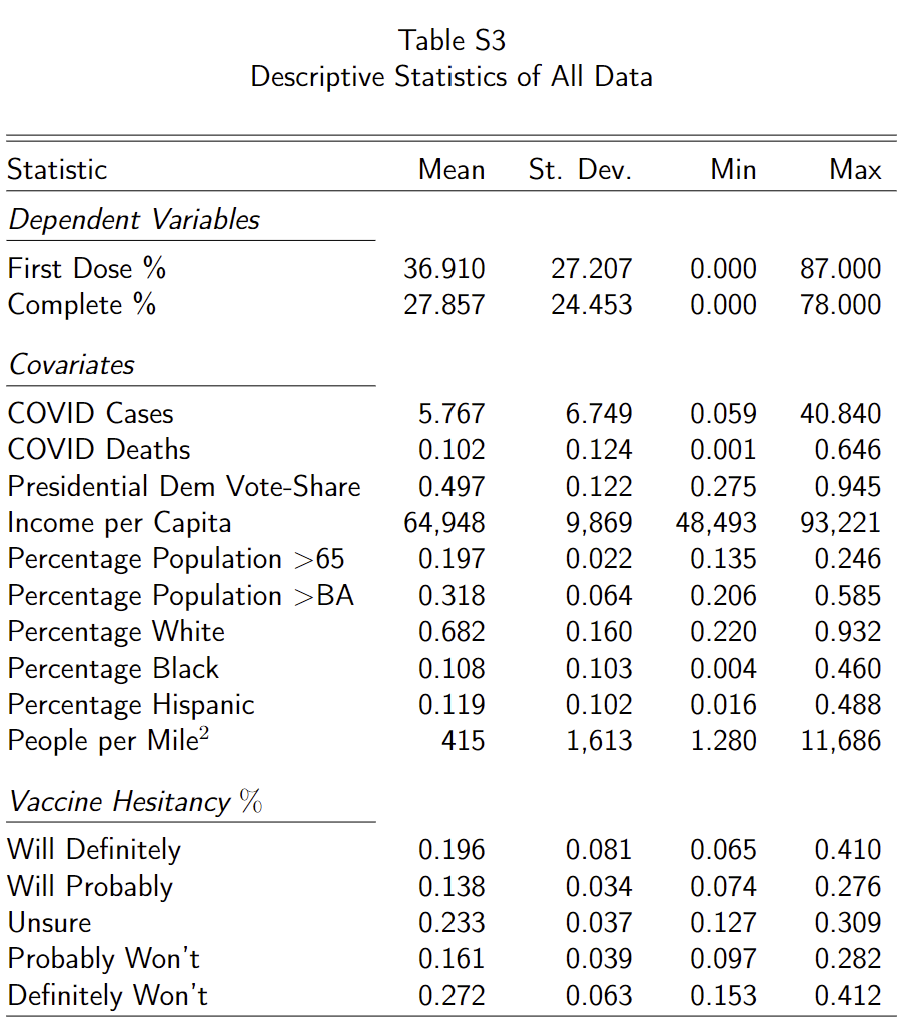

Supplement: S3 Table — (PNG) [file pone.0274374.s005.png]
